# Assessing good physical health and resilience as a foundation for positive welfare in chickens

**DOI:** 10.1016/j.psj.2026.106805

**Published:** 2026-03-17

**Authors:** Lisa Jung, Ruth C. Newberry, Yukari Togami, Manja Zupan Šemrov

**Affiliations:** a Institute of Science for Innovation and Sustainable Poultry Farming (WING), University of Veterinary Medicine, Hannover 30173, Germany; b Department of Animal and Aquacultural Sciences, Faculty of Biosciences, Norwegian University of Life Sciences, Ås 1432, Norway; c Department of Veterinary Sciences, Faculty of Veterinary Medicine, Ludwig Maximilian University of Munich, Munich 80539, Germany; d Department of Animal Science, Biotechnical Faculty, University of Ljubljana, Domžale 1235, Slovenia

**Keywords:** Positive animal welfare, Animal-based indicators, Laying hen, Broiler, Resilience

## Abstract

This review proposes candidate animal‑based indicators of good physical health and resilience in chickens (*Gallus gallus domesticus)* as a foundation for assessing positive animal welfare, complementing existing approaches to animal welfare assessment focused on use of iceberg indicators to detect severe health problems. We outline potential anatomical indicators involving the comb and wattles, eyes, beak, plumage, skin, footpads, claws, and overall body for rapid on-farm screening that could be automated for ease of application (e.g. using computer vision). We also identify health- and resilience-related anatomical and physiological indicators that could provide deeper, context‑dependent insights but require controlled testing conditions and/or laboratory analysis. For each indicator category, we summarize biological significance, influencing factors, and measurement methods under commercial and research settings. We classify candidate indicators according to their focus (health vs resilience) and response directionality on a scale from tolerable to optimal (whereby optimal values are highest for unidirectional measures such as plumage condition and intermediate for bidirectional measures such as claw length). We also rate potential ease of data collection (invasive, catching required, or remote sampling), on-farm applicability, and level of promise as a guide for indicator selection and prioritization for validation. Following validation and establishment of an appropriate scoring range from tolerable to optimal for each indicator depending on age, breed type, and reproductive status, we propose the use of continuous visual analogue scales or algorithms for scoring, followed by aggregation of indicator scores to obtain an overall rating of each bird’s health and resilience. This narrative review thus provides a biologically grounded roadmap for developing proactive assessment tools that support thriving in poultry, as a foundation upon which affective and cognitive components of positive animal welfare can also be added.

## Introduction

Animal welfare assessments have traditionally focused on negative mental and physical health states such as pain, fear, injury, and disease, with the aim of identifying the most severe problems and reducing their prevalence. However, the concept of animal welfare also includes the capacity to thrive and experience positive affective states (Zimmermann et al., 2011a, 2011b; Marino and Colvin, 2015; Webb et al., 2019). To facilitate the development of knowledge about the positive end of the animal welfare spectrum, Rault et al. (2025) have defined positive animal welfare (PAW) as a state of flourishing incorporating predominantly positive mental experiences, the development of competence (e.g., locomotor and social skills), and the capacity to recover from challenges (i.e., resilience). While Rault et al. (2025) emphasize that PAW goes beyond ensuring good physical health, a well-functioning body provides a foundation for positive mental experiences and skill development and is integral to resilience via homeostatic and allostatic processes. Homeostasis stabilizes internal conditions in response to immediate needs (Billman, 2020), while allostasis allows for anticipatory adjustments to environmental stressors (Korte et al., 2005; McEwen, 2016). Thus, good physical health can be viewed as a dynamic state of structural, physiological, and functional integrity supporting behavioral flexibility and emotional regulation (Martínez-Yáñez et al., 2025). In essence, good physical health is foundational to PAW for both immediate well-being and long-term quality of life. Within this broader animal welfare framework, physical health can be assessed by measurable signs of current physical health status, such as skeletal integrity and immune function. Resilience can be rated based on evidence of capacity to recover rapidly from typical stressors faced in daily life, and mental health can be evaluated using affective and cognitive indicators.

In chickens (*Gallus gallus domesticus*), efforts to assess and support PAW must accommodate flocks differing in age, production goals, management practices, and welfare challenges. However, all chickens share the need for good physical health and resilience to support adaptive functioning, the development of competence, and the potential for positive experiences. Anatomical and physiological indicators of good physical health and resilience thus offer an entry point for integrating PAW into welfare assessment frameworks. Anatomical measures are often relatively stable across contexts and easy to implement (Browning, 2022). This makes them particularly relevant when designing welfare assessments for on-farm assessments by producers or third-party welfare assessors, who require methods that are rapid, non-invasive, and easy to interpret. More complex physiological measurements may be used by researchers to investigate underlying mechanisms and validate new indicators.

At present, the field lacks validated animal-based indicators that reliably capture the range from tolerable to optimal physical health and resilience, as opposed to presence vs absence of severe pathology, or degrees of pathology. A tolerable state can be defined as a pathological state from which the animal can recover or a stable, sub-optimal non-pathological condition, and an optimal state can be viewed as the best possible state of individual bodily integrity, functionality and adaptability achievable within a particular age class, breed type and reproductive status. By switching the focus from severe pathology to the more positive end of the health spectrum, it is necessary to consider indicators of a multitude of healthy bodily functions that support thriving, rather than (at the negative end of the spectrum) focusing on a few iceberg indicators of severe loss of function.

In this narrative review, we integrate selected findings from the scientific literature to provide a thematic synthesis of how good physical health and resilience in chickens could be evaluated. We focus on potential animal-based indicators that can reflect structural integrity, functional capacity, and adaptability, considering how they can be assessed in live birds in commercial or research settings. The review is structured in two main sections: anatomical indicators, and physiological indicators. For each indicator category, we address its biological significance, briefly touch on factors that influence it, and consider available methods for assessment, resulting in a broad though non-inclusive catalogue of candidate indicators. We note that current methods mostly focus on pathology, and good health is inferred only by its absence, often making it necessary to report existing methods from this perspective as a basis for future work to define what constitutes a range from tolerable to optimal. We provide brief notes regarding indicator-specific interpretation of findings, with more general comments in the discussion section, and cite relevant articles for further details. After identifying candidate indicators, we then classify them according to whether they can provide a snapshot of current health status or serve as a marker of resilience, and whether they are unidirectional, with values rising from tolerable to optimal, or bidirectional, with tolerable values both below and above optimal intermediate values. We also rate each indicator according to whether its measurement requires catching of birds for invasive or non-invasive sampling or can be done without disturbing the birds, and whether it is feasible for on-farm use or requires specialized testing or laboratory analysis. Finally, we provide our estimate of each indicator’s level of promise, a judgement weighing factors such as predicted validity, sensitivity, specificity, relevance, and interpretability.

Our review does not propose a finalized assessment framework but, instead, provides a roadmap for selecting indicators for development and validation. As such, it sets the groundwork for moving beyond a primarily deficit-focused view of welfare assessment toward a model centered on health and resilience as a dynamic condition of vitality and adaptability. While we focus here on physical health and resilience as a complement to existing assessment efforts, it will be necessary to add candidate indicators of positive emotions and competence development for full PAW assessment.

## Anatomical health indicators from comb to claws

### Comb and wattles - ornaments with meaning

The comb and wattles are primarily comprised of connective tissue rich in blood vessels, covered by skin (Mukhtar and Khan, 2012). Vascularization gives them their characteristic red color, while collagen fibers add structure and flexibility. Both comb and wattles are androgen-dependent, with complementary functions as fitness-dependent indicators of disease resistance, health, and nutritional status (Mukhtar and Khan, 2012). Larger combs signal higher adult social status and play a role in preferential mate selection in both males and females, while female comb size has been correlated with the quantity of sperm deposited by males when mating (Cornwallis and Birkhead, 2007). Comb shape, and the side to which a single comb lops, provide additional information about individual identity (Guhl and Ortman, 1953) but whether these characteristics have significance for welfare is unclear (Holt et al., 2024a). Larger wattles increase conspicuousness and thereby enhance the signaling efficacy of behavioral displays, for example, by decreasing female orienting latency during the rooster tidbitting display (Smith et al., 2009). In addition, wattle symmetry of males was associated with higher egg production by their female mates in a line of Brown Leghorns (Forkman and Corr, 1996), although Bilcik and Estevez (2005) detected no link between wattle symmetry and measures of reproductive fitness in a commercial line of broiler breeders.

Comb and wattle color and size reflect the stage of reproductive maturation and readiness for egg-laying (Eitan et al., 1998; Mukhtar and Khan, 2012). From a thermoregulatory perspective, larger combs and wattles aid in heat dissipation during hot weather whereas smaller size is advantageous in cold climates by minimizing the risk of frostbite (Hester et al., 2015). An excessively large, lopped comb can hinder vision and smaller combs may sustain fewer peck wounds (Cloutier and Newberry, 2002). Overall, the optimal comb and wattle size in terms of biological functionality represents a balance between advantages and disadvantages of large size depending on environmental and social conditions.

**Fig. 1:**
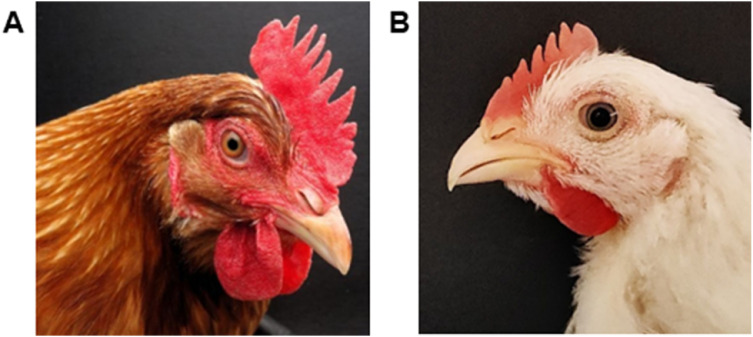
Healthy comb and wattles of (A) a laying hen (dual-purpose White Rock x New Hampshire, 36 weeks) and (B) a broiler (male, Ranger classic, 35 days).

The comb and wattles combine multiple features, including color, size, symmetry and skin condition, that are relevant for comparing the health of individuals within age, sex, genotype, and reproductive status. Assessment methods include visual inspection (e.g., Welfare Quality®, 2009), evaluation using photographic references (e.g., Blokhuis, 2007), and image digitization (e.g., Holt et al., 2024a). Typically, a healthy chicken exhibits a uniformly red comb and wattles mainly free from wounds or abrasions (Fig. 1). Conversely, pale, flaccid ornaments can indicate poor physical condition due to, for example, anemia, dehydration, or parasitism, although they can also indicate natural processes like molting. Comb perimeter length and relative bilateral symmetry of wattle size may indicate long-term developmental stability and ability to withstand allostatic load (Campo et al., 2010; Holt et al., 2024a). Within adult flocks, a combination of comb and wattle measures may provide a sensitive, integrated index of individual differences in health.

### Eyes, ears, and beak - elemental for sensory perception

***Eyes***. As precocial animals, the sensory systems of chickens are functional from hatch. As diurnal animals, chickens rely heavily on vision for essential behaviors such as foraging, navigating their surroundings, and distinguishing friends from foe (Porter et al., 2005). Their eyes provide an approximately 300-degree field of vision and excellent motion detection (Moore et al., 2022), with a small central binocular field used in pecking and individual recognition (requiring approach within 20-30 cm; Dawkins, 1995; Trapp and Fernandez-Juricic, 2022). Approach patterns, head movements and saccadic eye movements facilitate vision by gathering information via different specialized areas of the retina and lateralized functions of each eye (Pratt, 1982; Dawkins and Woodington, 2000; Rogers, 2010). Chickens have tetrachromatic vision, with four types of single cones sensitive to wavelengths ranging from around 370–700 nm (near ultraviolet to red), double cones thought to be sensitive to luminance, magnetoreception and polarized light, and rods that enable vision under dim light (Seifert et al., 2020; Chetverikova et al., 2022).

Environmental conditions, particularly light intensity and photoperiod, are critical for regulating circadian rhythms, feeding, mating behavior, and overall vision (Prescott et al., 2003; Egbuniwe and Ayo, 2016; Franco et al., 2022). High ammonia and dust levels can impair ocular health (e.g., Kristensen and Wathes, 2000), although chicken eyes are partially protected by a nictitating membrane that maintains corneal clarity and protects against environmental irritants (Jones et al., 2007). Nutrients such as vitamin A and carotenoids support retinal function and overall ocular integrity (Aydelotte, 1963).

**Fig. 2:**
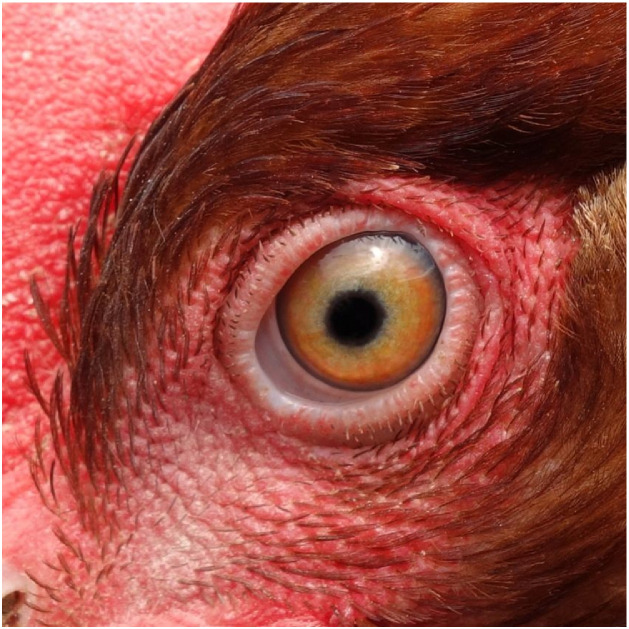
Clear and functional eye of a laying hen.

Several methods are available to assess ocular health in chickens and confirm normal, healthy eye function. Under farm conditions, eyes can be visually inspected for clear corneas, symmetrical eye diameter, and the absence of discharge or swelling (Fig. 2). Fact sheets provide practical recommendations for assessing ocular health (e.g., Lossie and McDermott, 2019). In veterinary and research contexts, several non-invasive ophthalmic diagnostic tools can be used. For example, Nardi et al. (2021) established reference values for healthy eye parameters in two chicken breeds based on tear production (measured via Schirmer tests, reflecting lacrimal gland function and ocular surface hydration), intraocular pressure (via tonometry, indicating fluid regulation and structural integrity of the eye), and ocular dimensions (using ultrasonography, providing information on eye morphology and developmental integrity) which together support the visual functioning required for engagement with the environment. Fluorescein staining can be used to confirm the integrity of the corneal surface, corneal clarity can be clinically graded, corneal topography can be mapped, and endothelial cell density (a measure of corneal functionality) can be determined from digitized images (Fowler et al., 2004). Behavioral tests can be used for assessing visual ability based on pecking a visual target on a touch screen (e.g. Sridharan et al., 2014).

***Ears***. The ears play an integral role in balance and sound detection, which is important for vocal communication, locating resources, and learning the significance of diverse environmental sounds. Chickens show a preference for consonant over dissonant sounds (Chiandetti and Vallortigara, 2011). They have high-resolution sound localization acuity, apparently attending to interaural time differences at low frequencies and interaural sound level differences at high frequencies (Krumm et al., 2022). At 60 dB, they are able to detect frequencies in the range of 9.1 Hz (infrasound) to 7.2 kHz (Hill et al., 2014).

Not only can a quiet environment avoid noise-related stress and fear responses (Campo et al., 2005), but it helps to maintain the integrity of the cochlea, reducing progressive hearing loss in adults (Durham et al., 2002). The risk of cochlear damage is minor in adults kept at 60 dB or less, although aging and genetics also play a role, and hearing loss is often more pronounced in one ear than the other (Smittkamp et al., 2002). However, progressive hearing loss is not inevitable, as birds have the ability to regenerate hair cells in their inner ear and recover hearing following damage (Langemann et al., 1999).

Although flock- rather than individual animal-based, the most practical on-farm method for estimating hearing ability would be to obtain sound level readings for comparison with validated data on hearing ability at different ages when kept under different noise levels starting from egg setting (as the auditory system becomes functional during incubation). In the laboratory, the ability of individual birds to hear specific frequencies can be assessed through behavioral testing in a sound-proofed chamber (e.g. Langemann et al., 1999). An estimate of ear health can be obtained through measurement of distortion product otoacoustic emissions, a procedure which detects the sound of cochlear outer hair movement when exposed to pure tones, reflecting cochlear cell integrity as a marker of hearing ability (Durham et al., 2002).

***Beak***. The beak is a vascularized and innervated bony projection of the jaws ensheathed in keratin. It supports the nares (nostrils) and tongue, and contributes to many vital functions including feeding, drinking, exploring, mating, nesting, preening, parasite removal, vocalization, and defense against predators and rivals. A straight beak, defined by symmetrical alignment of the upper and lower mandibles on the head’s median plane, is necessary for grasping functions and mouth closure. The nares are crucial for the sense of smell and the development of olfactory memories via olfactory receptors in the nasal epithelium. Odor detection contributes to attraction to familiar environmental stimuli associated with positive experiences, including foods and group members, and avoidance of noxious chemicals and odors associated with negative experiences (Roper, 1999; Krause et al., 2016; Papageorgiou et al., 2022). The nasal cavity cleans, warms, and humidifies the air before it enters the lungs, keeping harmful particles out of the respiratory system (Roper, 1999), while panting facilitates thermoregulation in hot weather. The mouth contains salivary glands, and taste buds in the gustatory epithelium of the palate, posterior area of the tongue, and base of the oral cavity (Cui et al., 2017) that enable detection of umami, bitter, salty, and sweet stimuli (Cheled-Shoval et al., 2017; Niknafs and Roura, 2018; Yoshida et al., 2021). The lower beak tip contains the bill tip organ comprising dermal papillae used for tactile discrimination, allowing birds to accurately feel and manipulate objects (Gentle and Breward, 1986; Iqbal and Moss, 2021) supported by actions of the tongue. An intact beak tip appears to be necessary for magnetoreception (Freire et al., 2011). Integrity of the beak tip is clearly important for beak functionality and avoiding pain from beak trimming (Hughes and Gentle, 1995; Janczak and Riber, 2015).

Beak condition depends on a balance between avoiding overgrowth (e.g., due to a pelleted diet requiring limited pecking) and avoiding excessive wear (e.g., when feeding requires extensive pecking on an abrasive surface). Beak straightness is influenced by a bird’s ability to cope with non‑genetic developmental disturbances such as incubator temperature fluctuations, unilateral eye malformations, beak trimming, infections, nutritional challenges, and beak overgrowth (Yamauchi et al., 2017; Joller et al., 2018). Ulceration and necrosis of the tongue and oral mucosa resulting from consumption of mycotoxin-contaminated feed (Vaccari et al., 2017) can be expected to impair taste and tactile sensations, while 6 weeks of exposure to 20 ppm ammonia (Anderson et al., 1964), and dusty feed caked over the nares, impair olfaction. In contrast, the ability to experience a rich variety of odors, tastes and textures may contribute to good health by stimulating neurogenesis, thus contributing to neuroplasticity (see Neuroplasticity section below). It has been found that detection of different odors stimulates neurogenesis in the olfactory system of chickens (Gomez and Celii, 2008; Hughes and Cunningham, 2017), a process likely extending throughout life.

**Fig. 3:**
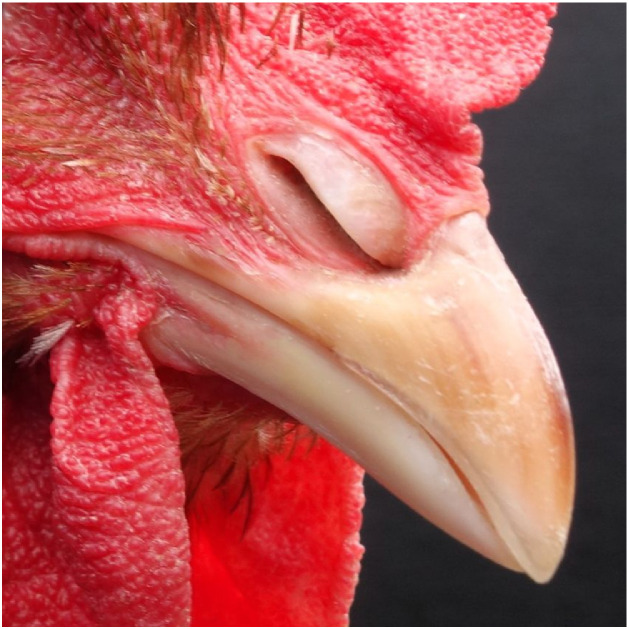
Laying hen with an intact beak and clean nares.

On the farm, chickens can be examined for an intact, straight, well-maintained beak with clear, unobstructed nares and oral cavity, and an absence of infraorbital sinus swelling, mouth and tongue lesions, allowing them to perform natural behaviors without pain or difficulty (Fig. 3). Beak length and symmetry can be quantified through morphometric assessment of digital images (Iqbal and Moss, 2021), whereby an angle of deviation of 0-2° between the beak axis and the cranial midline is considered straight (Joller et al., 2018). Using machine learning, infrared thermography has been used to distinguish respiratory health from respiratory distress in broilers (Elmessery et al., 2023). Behavioral testing can be used to assess behavioral responsiveness to odors and flavors as well as preferences when offered choices (e.g. Jones and Gentle, 1985; Burne and Rogers, 1999; Cheled-Shoval et al., 2017).

### Feathers and skin – first line of defense

The skin, composed of epidermis, dermis, and hypodermis, forms an outer protective layer that functions as a barrier against physical, chemical and biological harm. It contains mechanoreceptors, pruriceptors, and nociceptors, as well as thermoreceptors for detecting heat and cold. It also contributes to immune defense and vitamin D synthesis. Chickens lack sweat glands, but they possess a sebaceous gland above the tail, the uropygial gland, that secretes a waxy oil with hypothesized waterproofing, lubricating, antimicrobial, antiparasitic, antipredator, and social communication properties (Hirao et al., 2009; Moreno-Rueda, 2017). This oil is distributed through the feathers during the oiling component of preening behavior. The feathers, composed of keratin, are hierarchically branching ectodermal structures playing roles in communication, flight, and thermoregulation (Herremans et al., 1989; Chen et al., 2015). When structurally intact, clean, and well-oiled, feathers have protective properties that support PAW (Proudfoot et al., 1979; Peguri and Coon, 1993; Saraiva et al., 2016). Dustbathing behavior contributes to feather condition by fluffing the down and removing stale lipids and ectoparasites (van Liere, 1992; Martin and Mullens, 2012). Chickens naturally undergo an annual molt during which they shed and replace feathers (Zuberogoitia et al., 2018). While escape from danger is hampered, molting contributes to future welfare by replacing worn and damaged feathers.

Both genetic and environmental factors affect feather and skin integrity and resistance to feather deterioration, for example, by influencing the time spent active by broilers (Wilhelmsson et al., 2019). In laying hens, feather and skin integrity (i.e., intact plumage and skin) varies with factors such as breed (Schreiter et al., 2020; Malchow et al., 2022), pre- and post-natal stressors (de Haas et al., 2021), mash vs pelleted feed (Lambton et al., 2010), environmental enrichment (McAdie et al., 2005; Schreiter et al., 2020; Holt et al., 2024b), and use of an outdoor run (Bestman and Wagenaar, 2003). Leg (shank) skin integrity has been reported to be sensitive to wet/caked litter and ammonia, and to abrasive or poorly designed contact surfaces (e.g., perches), with higher stocking density increasing abrasion risk (Kristensen and Wathes, 2000; Bassler et al., 2013). Preen oil production and composition varies with genotype, sex, and plane of nutrition (Javurkova et al., 2023), while dustbathing effectiveness depends upon litter type (van Liere, 1992; Martin and Mullens, 2012), lipid content (Scholz et al., 2014), particle size, friability, and cleanliness (Moesta et al., 2008; Holt et al., 2023).

**Fig. 4:**
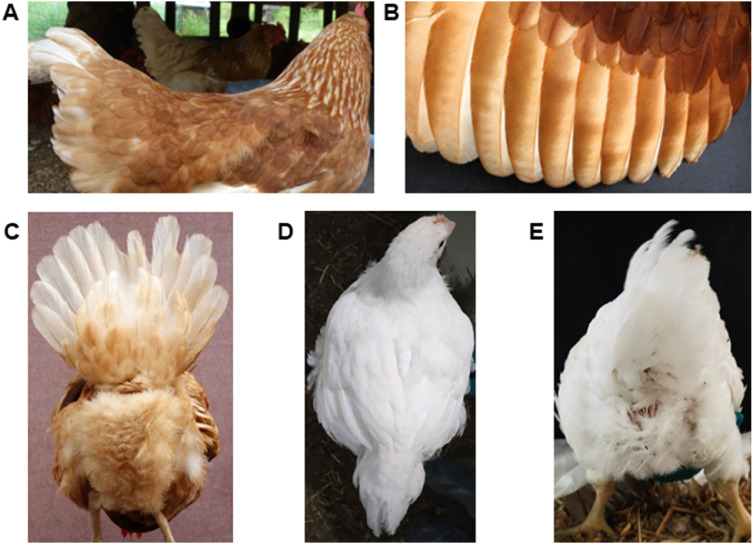
Clean, well-oiled, intact plumage of back, wing and cloaca/belly region of (A–C) a laying hen and (D and E) a broiler chicken.

The assessment of skin condition, plumage status, and cleanliness through visual inspection of individual birds is a widely utilized approach (Fig. 4). Protocols include, for example, LayWel (Blokhuis et al., 2007), simplified LayWel (Kjaer et al., 2011; Main et al., 2012), Welfare Quality® (de Jong et al., 2016), and transect sampling (BenSassi et al., 2019; Vasdal et al., 2023). Methods vary in resolution (e.g. scoring on a 5-point scale or as tolerable vs severe), whether the highest score is best or worse, time taken for completion, and whether or not the birds require handling. Inter-rater scoring reliability is generally good (Bright et al., 2006; Decina et al., 2019), though feather cleanliness scoring tends to be less reliable (Marchewka et al., 2013; Vasdal et al., 2022), possibly impacted by feather color, lighting conditions, bird posture and movement, and partial obstruction by other birds. Featherless leg (shank) skin provides a readily observable readout of integument health Khamas and Rutllant (2024), and can be scored for dryness/hyperkeratosis, erythema, abrasions or scabs. Computer vision methods for plumage assessment have been developed (Lamping et al., 2022; Zhang et al., 2024). Research methods include evaluation of plumage pigmentation and ultraviolet reflectance (Bright, 2007), preen oil composition (Alves Soares et al., 2024), and structural properties of the feathers such as fault bars (Jovani and Rohwer, 2017; Arrazola and Torrey, 2019) and fluffiness (van Liere and Bokma, 1987), all of which are relevant for feather functionality.

### Body size and weight – tracking optimal development

Body size and weight differ greatly between laying hens and broilers due to selection favoring egg and meat production, respectively, with dual purpose chickens being intermediate. Within these categories, there are also strain differences depending upon specific selection goals. For example, Lohmann Selected Leghorn hens typically have longer legs and a more streamlined body compared to Lohmann Brown hens. Among healthy birds, relatively large body size within a flock can serve as a status signal in adult social interactions, increase competitive ability, and lower the risk of cannibalistic attacks (Cloutier and Newberry, 2002).

Healthy growth depends upon nutrition, lighting conditions, and ability to maintain body temperature within homeostatic limits (e.g., Estevez et al., 2002; Baarendse et al., 2006; Jong et al., 2017; Raccoursier et al., 2019). However, selection for extremely rapid growth raises the risk of metabolic disorders and reduces adaptability (Castellini et al., 2016; Wilhelmsson et al., 2019). Regular monitoring of body weight allows for early detection of deviations from the expected growth pattern according to the breed standard and previous flocks in the same house so that corrections can be made before health issues become established.

Body weight is typically measured using calibrated electronic or mechanical scales, taking into account variations due to circadian patterns of feed intake. Morphometric measurements such as body length, shank length, and chest circumference can also be utilized to estimate body weight through predictive models (Obike et al., 2019). Body condition can be scored by palpation to assess the plumpness of breast muscle over the keel bone (Pedersen et al., 2020) or, for greater precision, by dividing body mass by morphometric measurements of body size (e.g. Clements and Sanchez, 2015). Automated weighing platforms and perches are commonly used for continuous, non-invasive weight tracking (e.g., Zhou et al., 2023). Precision may be improved by combining such systems with computer vision for body weight estimation (Campbell et al., 2025), and radio frequency identification tagging for individual weight tracking (Peng et al., 2022). Automated weighing systems require validation to ensure that the data are unbiased, comprise a representative sample of birds, and correctly capture individual weights (Newberry et al., 1985).

### The skeleton – supporting movement in three dimensions

The skeleton, composed of bones and cartilage, serves as the main structural framework of the body. It protects the internal organs, maintains mineral homeostasis, provides calcium for eggshell calcification, harbors bone marrow, and supports locomotion and flight (Rath and Durairaj, 2022). Depending on body mass, chickens retain a limited ability to fly short distances to perform motivated behaviors such as perching and using elevated nest boxes (Peters et al., 2016; Riber et al., 2018; Rentsch et al., 2023). Such physical activity exerts mechanical loading on bones, especially wing bones during flight, stimulating bone development and supporting welfare (Leyendecker et al., 2005; Regmi et al., 2015). The ability to fly depends on the functionality of the keel bone which anchors the muscles used for wing motion (Casey-Trott et al., 2015; Riber et al., 2018). The tail, consisting of a short section of fused vertebrae, the pygostyle, supports and controls tail feather movement used in flight (Rath and Durairaj, 2022).

In broilers, genetic selection heavily focused on rapid growth can impair skeletal health and associated activity and walking ability (Bergmann et al., 2017; Wilhelmsson et al., 2019). Diurnal rhythms are important for skeletal health, with a dark period increasing physical activity during the light period (Schwean-Lardner et al., 2012), and improving walking ability (Knowles et al., 2008; Bassler et al., 2013). Appropriate design and strategic placement of enrichment materials such as dust baths and perches may improve skeletal health and walking ability by stimulating activity (Ventura et al., 2012; BenSassi et al., 2019; Vas et al., 2023). In laying hens, bone health is related to activity, ossification (Toscano et al., 2020) and egg production variables (Gebhardt-Henrich and Frohlich, 2015; Eusemann et al., 2020; Malchow et al., 2022). The reported prevalence of keel bone fractures varies widely between populations, ranging from around 10 to 70% depending on strain, age, and management conditions (Jung et al., 2020; Rufener and Makagon, 2020; Kittelsen et al., 2021; Malchow et al., 2022).

**Fig. 5:**
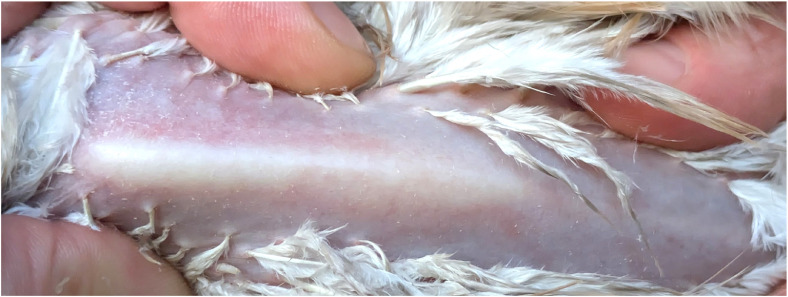
Smooth, straight keel bone of a laying hen.

Visual assessment of skeletal health can include body posture, gait symmetry, ease of walking, perching ability, and wing use during normal activities. The number of rotational corrections required to keep the body centered over the perch can provide a measure of ease of perching (LeBlanc et al., 2016). Standardized gait scoring systems are available for manual evaluation of locomotor ability (e.g., Welfare Quality^Ⓡ^, 2009), and automated continual monitoring on the farm is possible using computer vision based on optical flow (van der Eijk et al., 2023). “You Only Look Once” (YOLO) detection, tracking, and quantification of broiler walking speed from video images appears promising for real-time remote assessment of individual birds (Fodor et al., 2025; Jaihuni et al., 2025), as walking speed reflects manually assigned gait scores as influenced by pain (Tahamtani et al., 2021). It is unclear whether additional behavioural measures could improve sensitivity for detecting fine-grained differences in walking ability across a tolerable to optimal range, as well as enabling a distinction to be made between skeletal and other factors affecting walking ability (e.g. foot pad health, see below). Visual inspection and palpation, particularly of the keel bone, provides an indication of fractures, callus formation, and abnormal curvature (Casey-Trott et al., 2015; Fig. 5), while X-ray scans allow for more accurate and precise assessment (Emmert et al., 2024; Sallam et al., 2024). Given evidence of natural bone fracture healing in wild birds (e.g. forest-dwelling accipiters; Roth et al., 2002) as well as in commercial laying hens (Richards et al., 2011), there is a need to establish tolerable to optimal ranges for skeletal health in domestic laying hens of different ages, emphasizing functionality and capacity for recovery. In a research context, comparison of the relative length of left and right limb bones can be used to assess fluctuating asymmetry, whereby high symmetry indicates developmental stability and stress resilience. As asymmetries can be very small, precise measurements are needed. For live bird assessment, these are best accomplished using radiography (Van Nuffel et al., 2007).

### Footpad and toes – foundations of balance and grip

Chickens walk on their toes, making them the ballerinas of the barnyard, while fused foot and ankle bones form the tarsometatarsus (shank) that extends upwards from the footpad to the hock joint. Most chickens have three toes projecting forward and one projecting backward, although some breeds possess an extra toe at the back. The toes have two to four phalanges that facilitate fine-tuned balancing and grasping movements when standing, locomoting, manipulating objects, mating, and perching. They also contribute to the scooping of material during ground scratching, a component of exploratory (foraging) and comfort (dustbathing) behavior associated with PAW (Rayner et al., 2020). The toes are connected to leg muscles via flexor tendons arranged such that, when a chicken squats on a perch, the toes curl. With appropriately sized perches, this mechanical “perching reflex” allows chickens with healthy toes to sleep effortlessly on perches without falling off.

Multiple factors regarding genetics, housing, management, and early experience must be taken into account to minimize the risk of cannibalistic toe pecking behavior (Cooke, 1992; Buitenhuis et al., 2003; van Krimpen et al., 2008; Krause et al., 2011; Schreiter et al., 2020; Gebhardt-Henrich et al., 2023). Protection against cold is needed to avoid frostbitten toes, and equipment must be well-designed to avoid trapping of toes. Proper nutrition is important for toe development (e.g. adequate riboflavin to avoid “curled toe paralysis”; Ruiz and Harms, 1988). Rapid growth and rearing broilers in cages are risk factors for crooked toes (Rizk et al., 1980; Sanotra et al., 2001), which have been associated with an elevated heterophil:lymphocyte ratio (an indicator of stress; see below) and painful footpad dermatitis (Campo and Prieto, 2009). Foot damage also opens the door to bacterial infections and leg problems (Haslam et al., 2006). Footpad condition varies between laying hen hybrids (Schreiter et al., 2020), with cage-free hens being less likely to develop hyperkeratosis than hens in cage systems (Weitzenburger et al., 2006; Abrahamsson and Tauson, 2009; Blatchford et al., 2016), related to greater foot-related activity on varied surfaces and less time exposed to localized compression pressure from wire and perches. Acanthosis is also reported in caged hen footpads (Weitzenburger et al., 2006), potentially related to insulin resistance. In broilers, footpad condition tends to deteriorate with increasing age and body weight, with an associated reduction in activity (Gouveia et al., 2009; Bassler et al., 2013; Wilhelmsson et al., 2019), especially in fast-growing broilers (Sarica et al., 2014; Bergmann et al., 2016; Castellini et al., 2016; Wilhelmsson et al., 2019). Litter quality (clean, relatively dry, and friable) plays a crucial role in maintaining footpad (and hock) health (Bassler et al., 2013), which is influenced by stocking density, drinker type and management, and nutrition (e.g., Kamphues et al., 2014). These factors, along with precipitation and humidity levels on outdoor range, perch cleanliness (Jung et al., 2020), access to platforms (Tahamtani et al., 2020), and perch type and shape (Duncan et al., 1992) can contribute to differences in footpad condition (Gouveia et al., 2009; Sarica et al., 2014; Åkerfeldt et al., 2020; Jung et al., 2020).

**Fig. 6:**
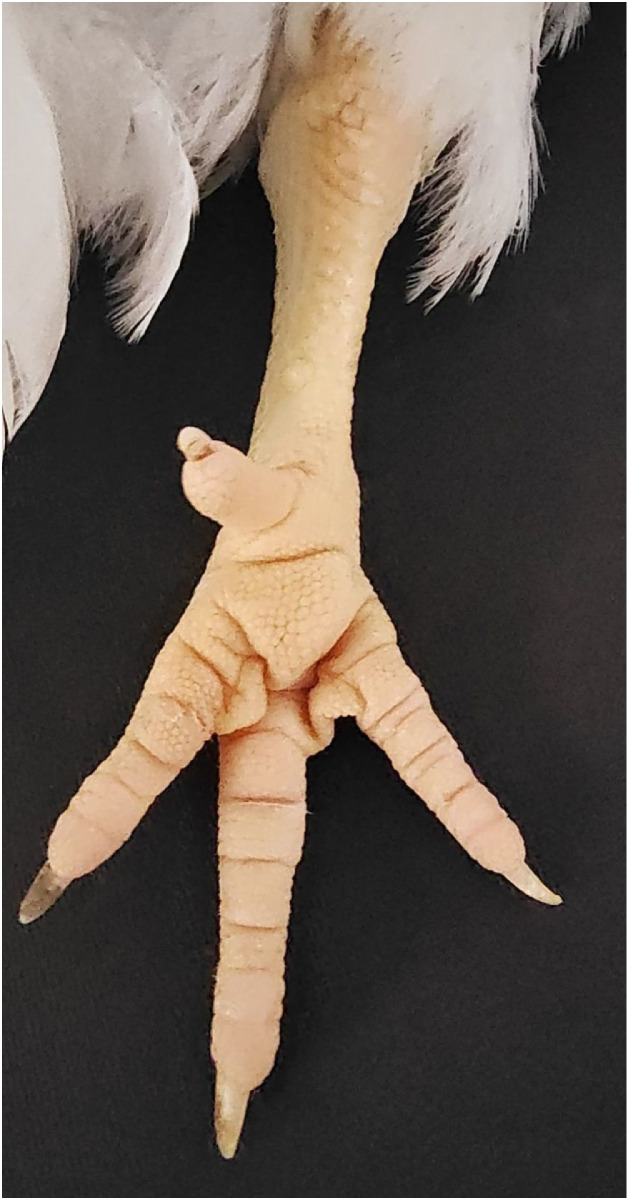
Broiler chicken foot, illustrating good footpad condition, intact toes, and claws of appropriate length.

Assessment of toe condition is primarily performed through direct visual inspection for freedom from abrasions, discoloration, swelling, deformities, and missing digits (Gebhardt-Henrich et al., 2023), while palpation aids in detecting subclinical issues such as stiffness or swelling. The footpads should have smooth skin free of cuts, scabs, ulcers, and abscesses (Fig. 6). Welfare Quality^Ⓡ^ (2009) provides a 5-point scoring system and sampling protocol for assessing the severity of footpad dermatitis, while simplified 4-, 3-, and 2-point systems are more practical for use on farms and at processing plants (Hunter et al., 2017; BenSassi et al., 2019; Meyer et al., 2020). Computer vision systems for automated footpad assessment during processing (Louton et al., 2022) overcome the need for live-bird handling and the challenge presented by dirty feet. However, proactive evaluation of foot health on the farm is more in keeping with a PAW focus that allows for recovery through early environmental adjustments. Indirect assessment of foot health based on computer vision movement tracking will likely become the gold standard (Dawkins et al., 2017; Bist et al., 2024b; Fodor et al., 2025), potentially aided by infrared thermography to enhance the detection of localized inflammation or early-stage pressure injuries (Bist et al., 2024a).

### Claws – multifunctional tools

Claws facilitate digging through the soil in search of food, an integral part of natural foraging behavior (Nicol, 2015). They enhance stability and grip during mating and perching (Appleby et al., 1992; Schrader and Malchow, 2020) and contribute to anti-predator behavior and self-defense (Khamas and Rutllant, 2024). Self-scratching is also used in body care. Similar to beaks, claws exhibit continuous growth and require natural wear through species-specific behaviors such as ground scratching and perching for optimal functional morphology (Schrader and Malchow, 2020).

Claw size in laying hens varies with housing system, with claws typically measuring 3–5 mm in width (Scott and MacAngus, 2004) and showing marked differences in length between systems. Overgrowth occurs in environments that restrict movement and lack abrasive surfaces (Tauson, 1986), such as in conventional cages where the middle claw may grow to 30 mm by the end of the laying cycle (Glatz, 2004). Shimmura et al. (2010) reported the longest claws in conventional cages (central front claws about 23 mm / hind claws about 13 mm), with progressively shorter claws in furnished cages (21–22 mm / 11–12 mm), single-tier aviaries (18 mm / 11 mm), and free-range systems (15 mm / 10 mm). Excessive claw elongation impairs locomotion and increases the risk of foot disorders (Knierim, 2006). Long, sharp claws can cause skin abrasions on flock mates, especially when scrambling for feed (Ruszler and Quisenberry, 1979) and during events triggering escape attempts (Honaker and Ruszler, 2004). Additionally, long claws can get trapped in housing equipment (Glatz, 2002; Vits et al., 2005). Use of claw shortening devices, such as abrasive strips that are scratched during feeding, can partially mitigate these risks by reducing claw length and sharpness (Glatz, 2002; Shi et al., 2019), but at a cost of increased cannibalism risk due to skin abrasion (Glatz, 2002). High litter moisture softens claws, making them more prone to breakage and infection, while excessive wear on hard or abrasive flooring can also damage claws (Tauson and Abrahamsson, 1994). White-feathered hens tend to have faster-growing, more abrasion-resistant claws, while brown-feathered hens exhibit more frequent ground scratching behavior, which naturally regulates claw length (van Emous, 2003). In broiler breeders, roosters with long claws can damage hens’ backs during mating, causing wounds and feather loss (Moyle et al., 2010). Broilers can also experience claw overgrowth related to inactivity and limited access to abrasive surfaces (Shields et al., 2005). Declawing poses significant welfare concerns by compromising foot support and stimulating neuroma formation, raising concerns about long-term pain (Gentle and Hunter, 1988).

Tauson (1986) measured claw length of the left and right middle toe with a tape measure and used a 4-point scale for assessing condition of the claw folds. Glatz (2004) used one 4-point scale to assess the extent of claw overgrowth, chipping and twisting, and another to assess claw sharpness by scraping the middle claw along a human fingernail and scoring the size of scratch produced. Shi et al. (2019) have developed a standardized imaging method to measure claw length and sharpness (curvature radius) in layer parent flock hens and roosters. Wu et al. (2024) fed real-time video images into a YOLO computer vision algorithm to detect the feet of caged laying hens on a commercial farm (https://youtu.be/f_NXkoem-bc), suggesting that automated detection of claw features should be feasible when cage-free birds are located on platforms and perches.

## Physiological indicators of health

### The immune system - the guardian of health

Immune responses play a vital role in maintaining chicken health. While immune responses are often associated with diseases and depressed behavior, reduced disease susceptibility (i.e., resistance or robustness) and faster recovery (i.e. resilience) facilitate PAW. Several studies in humans have shown that individuals with higher subjective well-being exhibit stronger immune responses (Huppert, 2009). However, this relationship is complex and context-dependent (D'Acquisto, 2017; Dupjan and Dawkins, 2022). In general, good health depends on the balance between the innate immune system characterized by immediate and nonspecific defense mechanisms and the adaptive immune system marked by generation of targeted antibodies and memory cells that confer long-term immunity (Berghman, 2016). Environmental stressors increase the ratio of heterophils to lymphocytes (H:L) and, when chronic, lead to negative health consequences due to persistent inflammation (Kaiser et al., 2009; Berghman, 2016). Levels of natural antibodies give an indication of inherited disease resistance (Sarrigeorgiou et al., 2023) with early immunity depending upon transfer of maternal immunoglobulins to the chick via the egg yolk. Secretory immunoglobulin A (IgA) is a key component of immune defense in the mucous membranes – particularly in the gastrointestinal tract – and serves as a first line of defense against pathogenic microorganisms. It has been proposed as a biomarker sensitive to changes in emotional valence that can be sampled non-invasively from feces, whereby higher levels have been associated with positive emotional states (Staley et al., 2018).

Multiple factors contribute to immunocompetence, including physical activity, sleep, short-chain fatty acids produced by the gut microbiota, omega-3 fatty acids and antioxidants from food, as well as limited environmental stressors (Dong et al., 2007; Hofmann et al., 2020; Wlazlak et al., 2023; Hofmann and Stefanski, 2024). For example, lowering the stocking density has been linked to greater bursa of Fabricius weight in broilers, which may reflect enhanced immune preparedness (Heckert et al., 2002). Environmental enrichment and social stability have been associated with relatively low H:L ratios in laying hens (Hofmann et al., 2020), as have natural periods of darkness allowing for undisturbed rest in broilers (Kim et al., 2022; Jiang et al., 2023). Environmental enrichment and low-density housing for broilers have also been associated with elevated levels of plasma and secretory IgA, respectively (Campbell et al., 2023).

Assessing H:L ratio, B- and T-cell populations and plasma immunoglobulins involves blood sampling, usually by venipuncture from the wing vein. To avoid potential changes related to handling stress, birds should be held in an upright rather than inverted posture and blood-sampled within 2 min of capture (Chloupek et al., 2011). Repeated measurements from the same bird are useful for evaluating resilience in response to challenges from specific stressors or antigens. Fecal samples can be collected easily for enzyme-linked immunosorbent assay for secretory IgA. However, interpretation of secretory IgA levels must take into account effects of age, diet, and circadian rhythms. Currently, assessment of immune functionality is restricted to veterinary and research purposes. Interpretation of immune measures is challenging as elevated immune activity may reflect either enhanced preparedness or ongoing immune challenge. Similarly, suppressed immune parameters can indicate reduced disease resistance but may also occur during energy reallocation under otherwise adaptive conditions. Interpretation therefore requires contextual information, repeated measurements, and consideration of prior antigen exposure. Interpretation could be enhanced through the development of a unified metric for immunological health in chickens as done for humans, for whom machine learning was used to distinguish healthy from unhealthy individuals based on a combination of whole blood transcriptomics, immune cell counts and hematological data (Sparks et al., 2024).

### The microbiome - more than a gut feeling

The gut microbiome is a complex system comprising bacteria, archaea, fungi, viruses, and their collective genomes, gene products and metabolites. It also includes the host’s genetic material interacting with these components in the gastrointestinal tract. The gut microbiome has a significant influence on the regulation of the host’s immune system, resistance to pathogens, metabolism, behavior and mental health (Stanley et al., 2012; Kogut, 2019; Chen et al., 2024), with functional pathways linked to gut-brain communication and metabolic resilience (Hu et al., 2022). The intestines of newly hatched chicks are almost free of microorganisms. Colonization begins through vertical transmission from the mother hen to her chicks or, in the case of artificial incubation, through horizontal transmission during handling and transport after hatching (Yue et al., 2024). It develops rapidly during the first 19 days after hatch and becomes relatively stable after 20 weeks of age. Health-associated characteristics of the microbiome include microbial evenness and enrichment with short-chain fatty acid-producing taxa (e.g., *Faecalibacterium*) (Hu et al., 2022).

Existing literature identifies diet composition (e.g. fiber and fermentable substrates), age, housing conditions, and antibiotic exposure as major contributors to gut microbiota composition and intestinal health in poultry (Apajalahti et al., 2004; Stanley et al., 2012; Oakley et al., 2014). High alpha diversity, with a stable, functionally rich microbial composition, has been associated with improved gut morphology, better nutrient absorption, and richer behavioral expression in chickens raised under welfare-promoting conditions such as free-range systems and enriched environments (Chen et al., 2018, 2019; Yan et al., 2020; Montoro-Dasi et al., 2021). de Jong et al. (2022) found differences in microbiome composition and diversity between broilers reared with and without dark brooder access from Days 0-14. Some of these differences lasted until depopulation age. Specific microbiota profiles, including elevated levels of *Lactobacillus spp.* and a higher Firmicutes-to-Bacteroidetes ratio, have been associated with improved feed efficiency and reduced fearfulness (Kraimi et al., 2019; Richards et al., 2020). These microbiome signatures consistently correlate with low fear responses, higher activity levels, and enhanced immunocompetence under low-stress conditions (Mohammed et al., 2021; de Jong et al., 2022; Chen et al., 2024). Demonstrating that these patterns remain detectable and meaningfully responsive to management improvements under typical commercial conditions would help confirm their value as indicators of good health.

Non-invasive microbiome evaluation is typically performed through 16S rRNA gene sequencing to assess the diversity and relative abundance of bacteria and archaea in ileal feces and cecal droppings (Alvarenga et al., 2023). For absolute quantification and profiling of metabolic pathways, whole-metagenome shotgun sequencing is used to sequence all DNA in the sample, including bacteria, viruses, fungi, and parasites (e.g. Durazzi et al., 2021). Defining the core chicken gut microbiota necessitates a focus on the stable microbiota of adult chickens (at least 20 weeks of age). While microbiome analysis provides valuable insights into gut health and host–microbiota interactions, interpretation remains challenging due to strong influences of age, diet, housing, and sampling procedures (e.g. fecal vs invasive cecal sampling; Kers et al., 2019), as well as limited consensus on what constitutes an optimal microbiome in chickens.

### Heart rate and heart rate variability - the rhythm of life

In chickens, resting heart rate (HR) typically ranges between 250 and 300 beats per minute in adult animals and between 350 and 450 in chicks (Detweiler and Erickson, 2004, cited in the MSD Veterinary Manual, n.d.). The HR is primarily regulated by the sinoatrial node and varies according to the body's physical demands. Elevated HR indicates arousal and energy expenditure, rising in response to both positively and negatively valanced environmental stimuli. This makes it unsuitable as a standalone indicator of positive emotional states (Waiblinger et al., 2006), but HR within a normal range is an indicator of good health when contrasted against abnormal HR and arrhythmia, a risk factor for sudden death syndrome in rapidly growing broilers (Olkowski et al., 2008). The speed of HR recovery to baseline after an acute event (e.g. egg laying, Mills et al., 1985) can provide an indication of resilience of relevance to PAW. Heart rate variability (HRV) refers to long-term variation in beat-to-beat intervals and can provide more precise information about the functional regulatory characteristics of the autonomic nervous system in response to psychophysiological stress (von Borell et al., 2007). Measured in ms, HRV provides an indication of the balance between sympathetic and parasympathetic nervous system activity. Lower HRV is generally associated with impaired autonomic flexibility, whereas higher variability within a normal range reflects greater regulatory capacity. Thus, relatively high HRV serves as an indicator of the emotional regulatory ability and adaptability of the autonomic nervous system (von Borell et al., 2007), although evidence from chickens is limited. A variety of HRV metrics are used to assess different aspects of autonomic activity (Shaffer and Ginsberg, 2017). For example, the Root Mean Square of Successive Differences (RMSSD) is based on normal heartbeats analyzed in 5-min epochs (i.e. after removing ectopic heartbeats and artefacts from the electrocardiogram). It emphasizes the contribution of parasympathetic activity (vagal tone) to HRV, which is especially relevant for PAW. The standard deviation of the intervals between normal heartbeats (SDNN) is typically measured over 24-h periods and emphasizes the sympathetic nervous system contribution to HRV in response to changing levels of activity.

In White Leghorns, Sturkie and Chillseyzn (1972) found that HR was highest at 2 and 4 weeks of age, thereafter declining to adult levels. In addition, HR was higher in females than males after 8 weeks of age. In broiler breeders, Savory et al. (2006) reported higher HR during the day than at night, especially in *ad libitum*-fed (vs feed restricted) birds. Ahmmed et al. (2023) confirmed the reduction in HR during the night in backpack-wearing White Leghorn pullets with implanted electrodes. This was accompanied by a drop in one measure of HRV, an increase in another, and no change in a third measure, indicating that care is needed in interpreting HRV findings. In White Leghorn hens with implanted heart monitors, Korte et al. (1998) found that the HR of hens from a low feather-pecking line tended to decline towards baseline more quickly after the onset of manual restraint than that of hens from a high feather pecking line, suggestive of greater stress resilience. These hens also had higher RMSSD readings during restraint, suggesting greater parasympathetic rebound following excitation of the sympathetic nervous system, which the authors equated to a “reactive” stress coping style as opposed to the more “proactive” stress coping style of hens from the high feather pecking line.

Electrocardiograms derived from implanted transmitters can provide reliable HR and HRV measurements in chickens, but these methods are invasive. Non-invasive wearable sensors are a promising alternative for long-term monitoring without restraint but require development and validation for welfare assessment in chickens. Systems based on optical sensing of blood flow may be impacted by skin color and obstruction by feathers. Other considerations when collecting data from devices attached to free-moving birds include battery weight and capacity, flock mate responses to the device, and the need for good sensor contact and comfortable fit to the bird (e.g. at different ages and during periods of high vs low activity). While both HR recovery time to baseline following an acute event and longer-term HRV assessment can provide information about cardiac resilience, their interpretation depends on baseline values, activity level, and circadian rhythms.

### Peripheral temperature - the chill factor

When the sympathetic nervous system is activated during acute emotional stress, vasoconstriction rapidly directs blood from the periphery to vital areas such as heart and skeletal muscle, resulting in a rise of core body temperature (stress-induced hyperthermia, SIH), accompanied by a brief drop in skin temperature followed by a return to baseline or, in more intense responses, a rebound above baseline (e.g., Edgar et al., 2013). SIH in laying hens and broilers reflects the degree of arousal when responding to both positive and negative challenges, and correlates with other commonly used indicators of stress including increased glucocorticoid levels, HR, and behavioral fearfulness scores (Moe et al., 2012; Edgar et al., 2013; Herborn et al., 2015, 2020). From the perspective of PAW, more relaxed, stress resilient chickens can be expected to show less exaggerated SIH and a more rapid return to baseline following an arousing event, or conditioned anticipation of one, thus being better prepared to respond to subsequent challenges and opportunities.

Herborn et al. (2020) found that chronic and intermittent stress was associated with elevated baseline comb temperature (fever), suggesting that cooler combs under baseline conditions may identify stress resilient birds. Consistent with this proposal, Ross et al. (2020) observed that hens living in an enriched environment had a lower baseline comb temperature than hens kept in more basic conditions. These hens also experienced a smaller drop in comb temperature than control hens when handled, and returned more quickly to baseline comb temperature (Ross et al., 2020). In a positive context, Moe et al. (2012) observed a drop in comb temperature when consuming mealworms, a highly rewarding food for chickens. Further, they detected an anticipatory decrease in the comb temperature of hens when presented with a conditioned cue signaling mealworm delivery. Soroko and Zaborski (2021) reported differences in mean beak temperature between genetic lines.

**Fig. 7:**
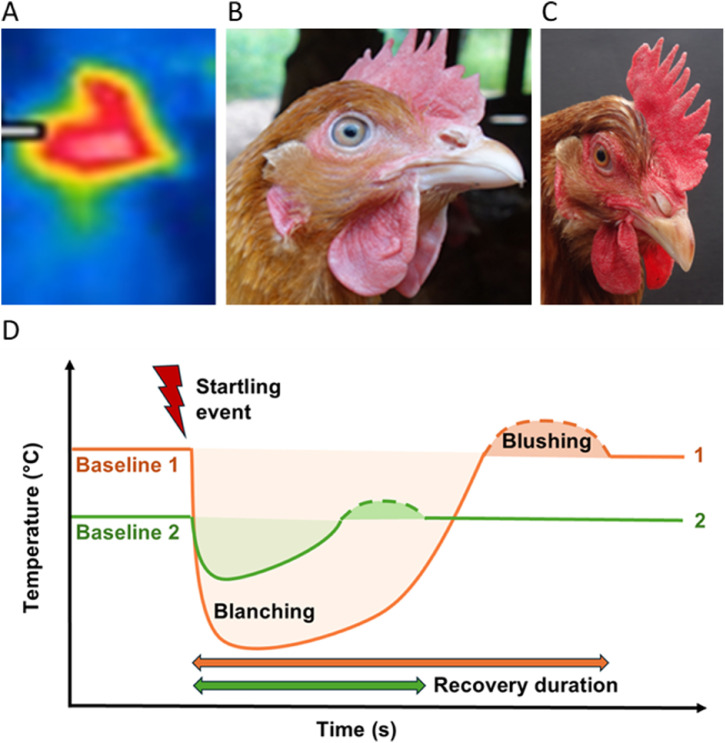
(A) Thermograph of a hen’s head (side view); (B) hen exhibiting peripheral cooling (blanching); (C) hen exhibiting “blushing”; (D) model of changes in peripheral temperature during stress-induced hyperthermia whereby bird 2 exhibits greater stress resilience than bird 1, with a lower baseline temperature, an attenuated stress response and more rapid recovery to baseline following a startling event.

Peripheral cooling and warming can be assessed by rapid sequential thermographic imaging of unfeathered and sparsely feathered body areas (comb, face, eyes, beak, lower legs, feet), a non-invasive method that assesses mid- to long-wave infrared radiation and presents it on a temperature scale (Tattersall, 2016; Soroko and Howell, 2018). As with HR, sufficient readings are needed to establish a stable baseline, detect maximal deviations from the baseline, and determine the time taken to return to a stable baseline while accounting for changes in baseline readings due to circadian and ultradian rhythms. The high vascularization of comb tissue makes the comb a particularly sensitive region for detecting changes in peripheral temperature (Fig. 7). In addition to temperature changes, changes in comb and facial color also reflect rapid changes in blood flow in response to emotional arousal. Importantly, the temporal dynamics of peripheral temperature changes provide additional interpretative value. A rapid return to baseline temperature following a stressor may indicate resilience whereas prolonged alterations may reflect compromised coping capacity. To capture changes in positive contexts, assessment of comb temperature and color should be done under undisturbed conditions, with repeated measures to capture fleeting changes in emotional status (blanching and “blushing”). When interpreting changes in peripheral temperature, it is important to take into account ambient air temperature (Ross et al., 2020), time of day, as well as increased core temperature resulting from physical activity (Edgar et al., 2013). There is some evidence that blushing may also result from emotional fever specifically during positive events (Arnould et al., 2024), possibly in the absence of SIH.

### Pupillometry - an eye on arousal

The iris:pupil ratio has been proposed as a standardized metric for evaluating physiological arousal in chickens (Mosco et al., 2025). In mammals, when the radial dilator muscle of the iris contracts during sympathetic activation, it pulls the inner edge of the iris outwards, causing the pupil to dilate, whereas when the circular sphincter muscle of the iris constricts under parasympathetic activity, the iris ringing the pupil is pulled inwards like a drawstring, resulting in a smaller pupil diameter. However, studies on birds are limited and it appears that pupil dilation can occur in deep sleep (Ungurean and Rattenborg, 2024), raising questions about the interpretation of iris:pupil ratio in birds.

Mosco et al. (2025) reported higher basal iris:pupil ratios in undisturbed laying hens located in outdoor vs indoor housing conditions, arguing that this measure reflects their physiological response to environmental quality. Thus, this measure might be relevant for health assessment. Existing reports on factors affecting the duration of changes in iris:pupil ratio in response to environmental events have focused on the response to changes in light intensity (e.g. Barbur et al., 2002), and it is currently unknown if response duration is relevant for assessment of stress resilience in general.

Evaluation of the iris:pupil ratio is a non-invasive method that can be achieved through digital image analysis of photographs of the eyes of unrestrained chickens, taken at eye level from a distance of at least 2 m (Mosco et al., 2025). Due to pupil constriction in response to brighter light, which can be affected by ambient light color (Barbur et al., 2002), controlled test conditions may be needed for reliable results. Further investigation is needed to assess the validity of this method for assessing good health, taking into account bird age, strain, behavioral context, and the accommodation response (i.e. constriction of the pupil to improve close-up vision). Serial sampling of changes in pupil size could be interesting for assessing resilience in response to different types and levels of acute challenge, though detecting small changes in unrestricted birds may be challenging. The magnitude of pupillary responses is illustrated by Barbur et al. (2002)’s observations on changes in adult laying hen pupil diameter in response to stimuli projected on a screen 30 cm away. Based on analysis of images from 50 Hz video recordings, they found that pupil size varied from about 5.3 mm under near-dark conditions to just over 4 mm in bright light. The pupil started to constrict in response to a flash of light in just over 50 ms (faster than the human eye), and showed pronounced constriction (over 0.3 mm) when birds were exposed to red but not green light at the same light intensity.

### Brain electrical activity - catching good waves

Electroencephalogram (EEG) activity reflects the synchronized activity of millions of cortical neurons, and can provide insights into cognitive and emotional states. The EEG of humans has been categorized into five main frequency bands—delta, theta, alpha, beta, and gamma, which have been associated with different mental states, from low frequency delta waves in deep sleep through theta waves in rapid-eye movement sleep, alpha waves in a relaxed but alert state, beta waves when decision-making, communicating, and focusing on external tasks, to high frequency gamma waves during high alertness and cognitive activity (Nayak and Anilkumar, 2024). Differences in EEG patterns within and across these frequency bands can predict self-reported positive vs negative mental states in humans (Lee and Hsieh, 2014). In chickens, EEG patterns become more complex and organized during embryonic development, reflecting increasing cortical integration and functional maturation of the brain circuitry (Hunter et al., 2000). Kollmansperger et al. (2023) concluded that embryonic day 13 marks the onset of significant EEG activity in chicken embryos, indicating the beginning of functional neural development necessary for nociception. Brain electrical activity in chickens follows a circadian rhythm primarily synchronized by environmental light–dark cycles, with the pineal gland and retina acting as key photoreceptors regulating neural and hormonal rhythms (Bernard et al., 1997). This circadian regulation is closely linked to sleep–wake patterns, melatonin secretion, metabolic processes, behavioral activity, and health and performance parameters in chickens (Geng et al., 2022; Bessei et al., 2023). Whereas healthy chickens show clear circadian rhythms, their circadian rhythms are blunted under stress, potentially resulting in a temporal mismatch between nutrient requirements and nutrient intake and increasing the risk for feather pecking and cannibalism (Bessei et al., 2023) and other adverse outcomes.

Because the EEG can be used to assess nociceptive responses with high temporal precision (Murrell et al., 2006), EEG studies in chickens have primarily focused on assessing the effectiveness of stunning procedures (Coenen et al., 2009). Nevertheless, there are opportunities for applying EEG to identify good health in chickens. For example, using surgically implanted EEG devices providing continuous data on successive nights, Putyora et al. (2023a; 2023b) established the baseline proportion of time spent by hens in slow wave sleep and rapid eye movement sleep, and the extent to which the hens’ sleep was disrupted by stressors varying in duration and intensity including noise, wind, light, high ambient temperature, feed restriction and footpad pain. Such data contributes to the understanding of two PAW-relevant features: quality of sleep and resilience to stressors.

**Fig. 8:**
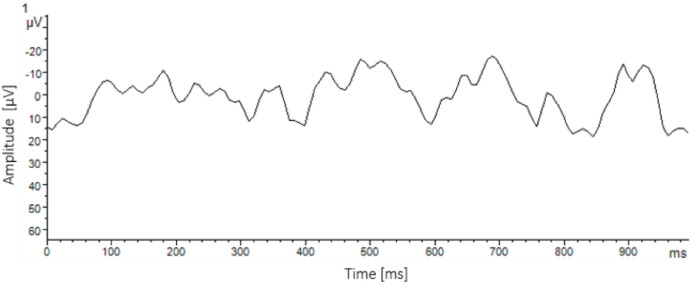
During a baseline measurement, electroencephalogram raw data from a male broiler chicken (Ross 308, 35 days) reveal predominantly alpha and beta wave activity. The x-axis shows time in milliseconds, and the y-axis shows amplitude of the signal (µV). From F. Kuck, Ludwig-Maximilians University, Munich DE following methodology of Togami et al. (2025).

Challenges in obtaining EEG data include the invasiveness of electrode implantation and difficulty in obtaining reliable readings under practical conditions (Verhoeven et al., 2015; Contreras-Jodar et al., 2022). To avoid the need for surgery, surface electrodes have been clamped, taped, or glued to the head (Coenen et al., 2007; Eberle et al., 2018; Togami et al., 2025) for measurements over brief time windows (Fig. 8). New non-invasive sensor technologies for remote recording coupled with algorithms for removing artefacts (e.g. muscular activity) would improve the feasibility of longer-term EEG assessment in free-moving, untethered chickens under research conditions, enabling analysis of circadian rhythms, sleep architecture, and proportions of time in different awake states in relation to environmental quality. On the farm, automated computer vision analysis of real-time video using a YOLO detector and tracking algorithm could be used to quantify the activity patterns of individual chickens over 24-h periods (Neethirajan, 2022; Yang et al., 2024), allowing assessment of the strength of their circadian rhythm and degree of synchronization with the photoperiod.

### Neurotransmitters - chemical sparks

Data linking neurotransmitters to health and resilience in chickens are limited. Nevertheless, neurotransmitters like dopamine, serotonin (5-hydroxytryptamine: 5-HT), endogenous opioids, mesotocin (the avian analogue of oxytocin), and endocannabinoids play important roles in regulating motivation, reward pathways, and emotional states in vertebrates (Bacque-Cazenave et al., 2020). In general, 5-HT supports social stability and emotional regulation, while dopamine promotes goal-directed behavior and learning by reinforcing pleasurable experiences (Dennis and Cheng, 2011). Dopamine motivates positive vocalizations (Riters, 2011), and has been linked to maternal care in chickens (Chokchaloemwong et al., 2015). By activating adrenergic receptors and engaging the hypothalamic–pituitary–adrenal axis, catecholamines such as adrenaline and noradrenaline contribute to long-term behavioral adaptations, which are central to coping strategies and resilience and therefore relevant for PAW (Dennis, 2016). Acetylcholine contributes to attention, memory, and stimulus recognition. Its dual role in the nervous and immune systems suggests broader regulatory functions relevant to health (Klinkenberg et al., 2011). Endorphins serve as natural analgesics (Scanes and Pierzchala-Koziec, 2018). Released during physically or emotionally challenging situations, they influence pain perception, avoidance learning, and emotional regulation (Clark et al., 1997). In this way, they represent an adaptive counterbalance to catecholaminergic stress responses. Riters (2011) points to the reward and satiety associated with the release of the endorphin, enkephalin, in the medial preoptic area of the avian brain. Mesotocin promotes affiliative behaviors in birds, especially in chicks (Loveland et al., 2019; Zhang et al., 2025). In mammals, its analogue, oxytocin, is known for facilitating bonding, social memory, maternal behavior, and trust, and complementing serotonergic and endorphin-mediated mechanisms (Lee et al., 2009; Ishak et al., 2011). Importantly, changes in neurotransmitter activity are not directionally specific with respect to welfare, as the same systems may be activated during both positively and negatively valenced experiences. For example, serotonergic, dopaminergic, and mesotocinergic pathways are involved in reward, affiliation, and emotional regulation, but are also responsive to stress, arousal, and social challenge, complicating interpretation of absolute levels.

Most investigations about neurotransmitters in chickens have focused on alleviation of negative states such as fear and aggression (e.g. increased dietary 5-HT reduced measures of fearfulness in Red Junglefowl hens; Lundgren and Lovlie, 2023). However, positive effects of 5-HT and dopamine on gut health and emotional regulation have been reported in broilers (Omaliko et al., 2024). From a study on the impact of genetic lines, brooding by a foster mother, and handling restraint on whole blood 5-HT levels, Rodenburg et al. (2009) suggested that peripheral 5-HT may serve as an indicator of stress sensitivity.

The suitability of neurotransmitters as markers for positive welfare is limited by their short half-life, the impact of handling to obtain samples, and the need to sacrifice birds to assess their activity in the brain. Analyses of blood and other secretions can provide data on peripheral neurotransmitter levels such as serotonin and adrenalin. While the measured levels are unlikely to directly reflect brain function, they may contribute to overall health assessment based on establishment of normal ranges, when interpreted in conjunction with other measures.

### Neuroplasticity - seeds of competence and resilience

Neuroplasticity includes fundamental biological processes such as neurogenesis that shape the brain’s capacity to adapt, learn, and respond to environmental challenges (Barnea and Pravosudov, 2011). Neurogenesis refers to the process by which new neurons form, migrate, differentiate, activate, and integrate into neural circuits. Their integration within the hippocampus is of particular interest for PAW due to the importance of this brain area for memory, emotion, and spatial navigation (Robertson et al., 2017). Neuroplasticity encompasses the brain’s broader ability to reorganize its structure, function, and connections in response to internal or external stimuli, such as learning experiences, stressors, or environmental enrichment (van Praag et al., 2000). In birds, including chickens, these dynamic processes are influenced by developmental conditions, social context, and environmental complexity (Armstrong et al., 2020). A brain that continues to grow and adapt reflects not just resilience to harm, but active engagement with the environment (van Praag et al., 2000; Barnea and Pravosudov, 2011). Neuroplasticity thus provides the neural substrate for adaptive responses to environmental change, contributing to PAW by enabling individuals to cope with challenges and develop competence. However, neural plasticity may also arise under conditions of environmental challenge or heightened cognitive demand. Interpretation therefore requires consideration of behavioral context, stress exposure, and functional outcomes such as cognitive flexibility and coping style.

Given that lateralized brain function has been associated with greater stress resilience, Kliphuis et al. (2024) measured proteins markers of neuroplasticity (calbindin D28k, doublecortin, neuronal nuclei protein) in the left brain hemisphere of laying hen chicks following incubation under daily 12-h green light exposure of the right eye vs continuous darkness. However, no treatment differences were detected. Armstrong et al. (2020) evaluated adult hippocampal neurogenesis in laying hens spending more vs less time on outdoor range. Based on levels of proliferating cell nuclear antigen mRNA, they found that hens spending more time on outdoor range exhibited higher levels of cell proliferation in the rostral hippocampus, suggesting an effect of cognitive enrichment and exercise when ranging outdoors. Higher expression of this marker in both the rostral and caudal hippocampus was also associated with longer durations of tonic immobility, suggestive of greater neurogenesis in hens with a more cautious but behaviorally flexible “reactive” (vs “proactive”) stress coping style. In contrast to these findings, hens spending the most time ranging in the open range area farthest from the house exhibited reduced expression of doublecortin in the caudal hippocampus, possibly related to greater stress exposure when visiting this area. van der Staay et al. (2012) argued that validated behavioral tests can serve as indirect but meaningful proxies for neural plasticity and brain health, making them valuable for assessing animal welfare. This proposal is supported by the finding that Red Junglefowl chicks with greater behavioral flexibility in a reversal learning task were also quicker to generalize and more fearful (Zidar et al., 2019), consistent with the “reactive” stress coping style of hens exhibiting greater adult hippocampal neurogenesis in Armstrong et al. (2020).

In chickens, neuroplasticity is typically assessed using a combination of molecular, histological, and immunohistochemical techniques that require access to brain tissue. One promising avenue is the development of peripheral biomarkers that can be sampled less invasively, such as the neurotrophic factors, brain-derived neurotrophic factor (BDNF) and nerve growth factor (NGF). These proteins play roles in neuronal survival, synaptic plasticity, and cognitive function and have been found in peripheral tissues, including blood (Lommatzsch et al., 2005). Another promising route involves neuroimaging techniques, such as functional near-infrared spectroscopy (fNIRS) and functional magnetic resonance imaging. Although challenging in chickens due to anatomical and technical constraints, portable and animal-friendly fNIRS systems have shown feasibility for use in other small animals and could, with refinement, be adapted to birds (Boas et al., 2014; Paik et al., 2021). Behavioral tasks such as spatial memory, detour learning, and reversal learning are increasingly used to assess cognitive flexibility and infer neuroplasticity in animals. Further research linking behavioral, physiological, and brain data is needed to validate these tasks for use in chickens.

### Telomere length - happy life clock

Telomeres consist of non-coding TTAGGG DNA repeats that protect the ends of eukaryotic chromosomes by preventing chromosome fusion and degradation (Barrett and Richardson, 2011). Telomere erosion is driven by oxidative stress and incomplete replication during cell division. As cell division is most pronounced during early life, environmental conditions during this period can significantly influence the rate of telomere attrition and, consequently, the rate of cellular aging (Foote et al., 2011). Whereas telomere shortening indicates stress and aging, longer telomeres have been associated with positive mood and mitigation of negative experiences in humans (Bateson, 2016). Also in humans, positive lifestyle interventions such as mindfulness meditation have been associated with increased telomerase activity, the enzyme responsible for telomere repair (Schutte and Malouff, 2014).

Despite a more compact genome, birds have five to 10 times more total telomere sequences than humans (Delany et al., 2000), and telomere length at hatch is maternally (vs paternally) inherited (Sohn and Subramani, 2014). In wild birds, whole blood telomere length has been correlated with measures of fitness depending on species and sex (Pauliny et al., 2006). Red blood cell telomere length has been found to decline with age in laying hens, accompanied by accumulation of nuclear and mitochondrial DNA lesions (Makarenko et al., 2017). Compared to control birds, broiler chickens fed a corticosterone-enriched diet exhibited telomere shortening in whole blood, muscle, liver and heart cells (Badmus et al., 2021). Moreover, broilers housed at a stocking density of 0.116 m²/bird had longer lymphocyte telomeres than those housed at 0.058 m²/bird (Beloor et al., 2010). In a study by Campbell et al. (2025), broilers housed in an enriched environment divided into functional zones and containing a dust bath, perching structures, pecking stones, and rotating enrichment items tended (P = 0.05) to have longer kidney (but not gonadal) telomeres than those housed in an environment lacking these enrichments. Stocking densities ranging from 22.1 kg/m² to 42.6 kg/m² did not affect telomere length in the tissues measured in that study.

Telomere length is measured in different cell types (including buccal cells for minimally invasive sampling), with results varying depending on cell proliferation rate. Common methods for measuring telomeres include quantitative real-time PCR, analysis of terminal restriction fragments, single telomere length analysis, and (most commonly) fluorescence in situ hybridization (Bateson, 2016). Because absolute telomere length is strongly influenced by age, tissue type, and cell turnover, telomere measures are most informative when used repeatedly to assess within-individual rate of biological aging over a specific period (Sohn and Subramani, 2014).

## Discussion

This overview highlights a necessary paradigm shift in animal welfare science: moving beyond a predominantly negative framework, focused on pathology, dysfunction, and disease prevalence, towards one that values indicators of good physical health and resilience. While traditional assessments emphasize impairment, we have explored candidate indicators that could be assessed across gradations of biological functioning in chickens ranging from tolerable to optimal, as a foundation for PAW. We propose use of indicators in an assessment framework that involves (i) selecting complementary indicators spanning acute arousal, short‑term regulation, and long‑term baseline health; (ii) repeated measurements to establish individual baselines and resilience; and (iii) interpreting patterns across multiple indicators. Table 1 synthesizes candidate indicators reviewed in this manuscript and organizes them according to focus (health or resilience), response scale (uni- or bidirectional), ease of data collection, on-farm applicability, and overall level of promise, allowing them to be assembled into context‑specific indicator sets. The classifications in Table 1 help to distinguish indicators that may be suited for rapid development, validation and routine application (e.g. skin and plumage integrity, walking and balancing ability, beak length and straightness, body weight trajectories) from those more suited to in-depth analysis in a research context (e.g. immune parameters, microbiome characteristics, telomere length, markers of neuroplasticity).

**Table 1:** Overview of potential animal-based indicators identified in this review and their main characteristics.

^1^Category	Candidate indicator	²Focus (health/ resilience)	³Response scale (uni- /bidirectional)	^4^Data collection (difficult, moderate, easy)	^5^On-farm applicability (yes/no)	^6^Promise (low, moderate, high)
**Comb & wattles**	Size	health	bidirectional	easy	yes	moderate
	Color	health	bidirectional	easy	yes	moderate
	Wattle length symmetry	resilience	unidirectional	easy	yes	moderate
	Skin integrity	health	unidirectional	easy	yes	high
**Eyes, ears & beak**	Clear corneas	health	unidirectional	easy	yes	high
	Eye diameter symmetry	resilience	unidirectional	easy	yes	moderate
	Reaction to visual stimuli	health	unidirectional	easy	no	moderate
	Tear production	health	bidirectional	moderate	no	moderate
	Intraocular pressure	health	bidirectional	moderate	no	moderate
	Hearing ability	health	unidirectional	easy	no	high
	Intact beak	health	unidirectional	easy	yes	high
	Beak length	health	bidirectional	easy	yes	moderate
	Beak straightness	resilience	unidirectional	easy	yes	high
	Tongue condition	health	unidirectional	moderate	no	moderate
	Clear nares	health	unidirectional	easy	yes	high
	Infraorbital sinus status	health	unidirectional	moderate	no	high
	Chemical sensory ability	health	unidirectional	easy	no	moderate
**Feathers & skin**	Plumage condition	health	unidirectional	easy	yes	high
	Feather cleanliness	health	unidirectional	easy	yes	moderate
	Leg skin condition	health	unidirectional	easy	yes	moderate
**Body size & weight**	Body weight	health	bidirectional	easy	yes	high
	Body condition	health	bidirectional	easy	yes	high
	Growth rate	resilience	bidirectional	easy	yes	high
**Skeleton**	Leg skeletal condition	health	unidirectional	moderate	no	high
	Walking ability	health	unidirectional	easy	yes	high
	Keel bone status	resilience	unidirectional	moderate	no	high
	Wing skeletal condition	health	unidirectional	moderate	no	high
	Flying ability	health	unidirectional	easy	yes	high
	Balancing ability	health	unidirectional	easy	yes	high
	Limb bone length symmetry	resilience	unidirectional	moderate	no	moderate
**Footpads & toes**	Intact toes	health	unidirectional	easy	yes	high
	Footpad status	health	unidirectional	easy	yes	high
**Claws**	Claw length	health	bidirectional	easy	yes	moderate
	Claw sharpness	health	bidirectional	easy	no	moderate
**Immune system**	Secretory immunoglobulin A	health	bidirectional	easy	yes	moderate
	H:L ratio	health	bidirectional	difficult	no	high
	B- and T-cell populations	health	bidirectional	difficult	no	moderate
**Microbiome**	Alpha diversity	health	bidirectional	easy	yes	high
	*Lactobacillus spp.* level	health	bidirectional	easy	yes	moderate
	Firmicutes:Bact- eroidetes ratio	health	bidirectional	easy	yes	moderate
**Heart Rate**	Baseline heart rate	health	bidirectional	moderate	no	moderate
	Heart rate recovery to baseline	resilience	unidirectional	moderate	no	high
	Heart rate variability	resilience	bidirectional	moderate	no	high
**Peripheral temperature**	Baseline comb temperature	health	bidirectional	easy	yes	moderate
	Comb temperature recovery to baseline	resilience	unidirectional	easy	yes	moderate
	Blushing	resilience	bidirectional	easy	yes	moderate
**Pupillometry**	Iris:pupil ratio baseline	health	bidirectional	easy	no	moderate
	Iris:pupil recovery to baseline	resilience	unidirectional	easy	no	moderate
**Brain electrical activity**	Circadian rhythm	health	bidirectional	easy	yes	high
	Sleep	health	bidirectional	moderate	no	moderate
**Neuro- transmitter pathways**	Neuro- transmitter levels	health	bidirectional	difficult	no	low
**Neuro- plasticity**	Hippocampal cell proliferation	health	bidirectional	difficult	no	moderate
	Brain-derived neurotrophic factor	health	bidirectional	difficult	no	moderate
	Reversal learning ability	resilience	unidirectional	easy	no	high
**Telomeres**	Telomere length	resilience	unidirectional	difficult	no	moderate

**Fig. 9:**
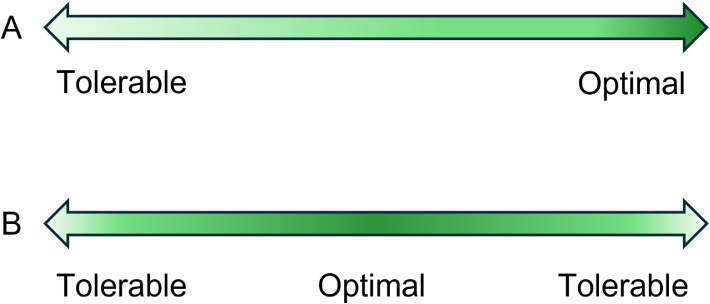
Visual analogue scale for assessing indicators of good health and resilience. (A) scale for unidirectional indicators where the optimal state has the highest value (e.g. plumage cleanliness, walking ability); (B) scale for bidirectional indicators, where the midpoint represents the optimal level (e.g. claw length, heart rate variability).

1Category refers to the corresponding section of the manuscript. ²Focus indicates whether the indicator primarily reflects a snapshot of a current health-related state or the bird’s capacity to cope with stressors and recover towards its baseline condition (i.e. resilience). ³Response scale (uni-/bidirectional) specifies whether indicator values vary from tolerable to optimal on a unidirectional or bidirectional scale, as illustrated in Fig. 9. ^4^Data collection reflects the extent to which birds must be disturbed to collect data (difficult: invasive sampling required; moderate: catching/handling required; easy: potential for automated sampling from undisturbed birds). When multiple sampling methods are possible, the score refers to the easiest potential solution (e.g. computer vision analysis of video camera input). ^5^On-farm applicability reflects the estimated feasibility of measuring or implementing the indicator under practical farm conditions as opposed to research conditions. ^6^Promise represents author consensus regarding the indicator’s overall potential validity, sensitivity, specificity, interpretability, relevance, and usefulness based on available evidence.

Anatomical indicators can be particularly valuable when combining biological relevance with practical feasibility. External observability and their potential for assessment with minimal or no handling of birds makes them suitable for routine on-farm assessments, either for manual snapshot assessments or continuous AI-based monitoring. Variables such as plumage condition and walking ability can be evaluated through visual inspection, lending themselves to structured scoring such as used in the Welfare Quality® and LayWel protocols (Blokhuis et al., 2007; Welfare Quality®, 2009). Existing practical frameworks, such as the Key Welfare Indicators for Broilers and Laying Hens (International Poultry Welfare Alliance, 2025) indicate that commercially feasible approaches already exist and can be expanded to include unidirectional metrics with gradations on the positive end of the assessment scale, and bidirectional metrics with optimal values in the center of the scale (Fig. 9). These indicators can be combined to characterize graded levels of biological functioning, resilience, and optimality. In contrast, physiological indicators present greater methodological and interpretational complexity, currently limiting their use to research contexts. Some measures, such as telomere attrition, provide insights into long-term welfare trajectories, whereas others (e.g., secretory IgA, HRV, peripheral temperature changes, and markers of neuroplasticity) can reflect health and resilience on shorter timescales. However, their interpretation is challenging due to the influence of multiple factors such as age, microbiota composition, infection status, and circadian rhythms. Many physiological indicators also require repeated sampling to assess stress resilience alongside individual baseline calibration. They are sensitive to context. For example, elevated immune activity may indicate either healthy stimulation or pathological stress. These challenges highlight the need to triangulate physiological measures with anatomical, behavioral, and environmental information to ensure biologically meaningful interpretation. Consistent with this complexity, few studies have evaluated diagnostic metrics such as sensitivity or specificity for either anatomical or physiological indicators in the context of PAW, underscoring the need for careful validation before broader application.

Farmers often remain hesitant about welfare self-assessment systems due to time and cost constraints (Michaelis et al., 2024). A commonly cited barrier is the additional labor required, which challenges the long-term feasibility of such systems in commercial settings. Integrating smart farming technologies and artificial intelligence (AI) could help mitigate this burden by enabling continuous, automated monitoring, supporting early detection of welfare issues and more precise interventions (Dawkins, 2025). However, when data are limited or non-representative, AI tools risk overfitting to narrow conditions and producing misleading classifications (Shwetha et al., 2024). Therefore, algorithms must be statistically grounded and transparent (Dawkins, 2025). Although they cannot substitute for good husbandry, when properly validated, AI-based tools allow for large-scale data collection, long-term tracking, and internal or external benchmarking of welfare outcomes (Jung et al., 2024). Internal benchmarking enables farms to monitor their own improvements over time, while cross-farm comparisons, with privacy safeguards to preserve anonymity can help define realistic and aspirational welfare targets. The effective use of such technologies will depend on their practical feasibility, ease of interpretation, and clear alignment with biologically meaningful indicators such as those outlined in Table 1.

Once indicators have been validated, graded assessment scales could be based on standardized five-point ordinal scales similar to existing manual scoring approaches (Blokhuis et al., 2007; Welfare Quality®, 2009). This format is practical, time-efficient, and accessible across varying levels of assessor expertise. In controlled research or testing contexts, or when using continuous data from real-time AI-based systems, visual analogue scales (VAS) can capture more fine-tuned gradients in bird condition (Fig. 9). Studies on negative animal welfare indicators in broilers have shown agreement between assessors using either ordinal or VAS scales as well as agreement between scales at the individual‑bird level (*r* = 0.77–0.95; Souza et al., 2024). For presentation of results VAS color gradients may enhance interpretability by intuitively signaling increasing levels of positive welfare, paralleling affective‑state visualizations such as heatmaps. Implementation of VAS scoring would require inter-rater calibration and digital tools for measurement and color scaling, and both scoring formats would require piloting to assess their reliability, ease of use, and suitability for different contexts.

To develop reliable assessment tools and welfare recommendations, it will be essential to define what constitutes an optimal state for each indicator, and to specify the range of the scale by establishing the extent of deviations from the optimal state that fall within tolerable limits. For physiological indicators such as HRV or secretory IgA, clinical reference ranges can be used if available, considering differences across breed, age, and reproductive status. However, for all indicators, it will be necessary to explicitly define what constitutes a tolerable to optimal range based on biological function. Importantly, depending on the indicator, optimality can occur on either a uni- or bidirectional scale. For instance, both excessively long and overly short beaks or claws can compromise function. Accordingly, clear thresholds are needed to determine when an indicator falls outside a tolerable range. Fraser (2008) describes optimality as the best achievable trade‑off among competing biological demands under given environmental conditions, a definition that emphasizes context without permitting compromised health to be normalized.

PAW is multidimensional, encompassing physical integrity, emotional expression, competence building, and adaptive capacity. Accordingly, no single indicator can adequately capture this condition. Instead, different indicators provide complementary information about the animal’s physical functioning and adaptive capacity. As summarized in Table 1, the candidate indicators are evaluated with regard to their potential usefulness, whereby some primarily reflect acute health status, whereas others may describe longer-term characteristics related to resilience. Taken together, indicators reflecting acute health states and those describing longer-term structural or functional characteristics can provide a more complete picture of an animal’s condition and its capacity to maintain stable functioning over time. Such conditions form an important basis for the expression of positive emotions and broader aspects of flourishing that are central to PAW. Hence, when developing complete PAW assessment tools, it will be necessary to integrate information regarding anatomical traits (e.g., plumage condition), physiological markers (e.g., secretory IgA, HRV), as well as cognitive/affective indicators (Mellor, 2016), into a cohesive welfare profile for each individual, enabling targeted individual-bird welfare interventions when needed. Because welfare is a dynamic state of balance and trade-offs between biological systems, not all indicators can be expected to remain continuously at optimal levels. Thus, while establishing indicator-specific thresholds to ensure that no domain falls below a tolerable minimum, a composite welfare assessment system can allow limited compensation between indicators. This approach would enhance comparability across environments and populations, and improve communication across scientific, regulatory, and producer stakeholders.

To bridge the gap between conceptual frameworks and practical implementation, future research should prioritize the validation of promising indicators that reflect real-world management variation. This includes establishing baseline values, assessing sensitivity to environmental enrichment and other welfare-relevant inputs, and evaluating robustness across breeds, life stages, and housing systems. Non‑invasive, repeatable indicators suited to routine longitudinal monitoring (e.g., fecal secretory IgA, body weight trajectories) should receive particular attention, as longitudinal data are essential for determining temporal sensitivity and capturing dynamic welfare trajectories. Consistency and standardization in measurement are also critical for credibility and comparability (Wemelsfelder et al., 2001), and, as emphasized by Knierim and Winckler (2009), protocols must be rigorously tested for reliability, validity, and feasibility to support meaningful welfare outcomes. Thus, systematic validation studies are needed to quantify the diagnostic and predictive value of these candidate indicators and to establish how they perform—alone and in combination—across graded welfare-relevant conditions. Practical recommendations for users can be made once these indicators have been empirically validated.

Ultimately, integrating PAW into animal management will require a conceptual and methodological shift, from detecting severe harm to identifying and supporting flourishing. When interpreted dynamically and in context, health-related indicators can anchor this transition by reflecting biological integrity and resilience. While this paper has focused on physical health and resilience as foundational for PAW, flourishing also depends on emotions and development of competence, often assessed through behavioral expression. As Rault et al. (2025) emphasize, true welfare encompasses affective well-being, intrinsic motivation, and social fulfillment, dimensions that reflect the animal’s own subjective experience. This paper provides a basis for advancing toward this broader vision, outlining how physical health and resilience indicators, when interpreted in an integrated, dynamic, optimality framework, can serve as the foundational layer of a comprehensive, biologically grounded model of thriving in poultry, one that connects physical health with cognitive, emotional, and social well-being.

## Conclusions

This narrative review highlights the importance of reframing a broad spectrum of health-related states, from external features such as feather condition to measures of internal parameters such as immune function, microbiome diversity, thermoregulation, neurophysiological activity, and telomere dynamics, as candidate indicators of physical health and resilience in chickens. However, each indicator must be interpreted in a time- and context-specific manner, acknowledging that only in combination can these diverse measures provide a reliable overall picture of a chicken’s capacity to cope, adapt, and thrive. While we propose practical tools for on-farm monitoring and encourage the use of validated, non-invasive approaches, the candidate indicators, as well as suggested VAS scaling, still require rigorous evaluation regarding their reliability, validity, and practicality. By reviewing and classifying candidate indicators, we offer both a conceptual roadmap for proactive assessment and a research agenda for further development. Ultimately, recognizing and promoting good health and resilience is not only a scientific endeavor but also an ethical and societal imperative. By integrating anatomical and physiological indicators within a PAW framework, this review provides the biological foundation for measuring not only how chickens survive, but how they thrive.

## Funding

No external funding was received for this study.

## CRediT authorship contribution statement

**Lisa Jung:** Writing – original draft, Visualization, Methodology, Conceptualization. **Ruth C. Newberry:** Writing – review & editing, Writing – original draft, Visualization, Methodology, Conceptualization. **Yukari Togami:** Writing – original draft, Visualization. **Manja Zupan Šemrov:** Writing – original draft, Supervision, Methodology, Conceptualization.

## Disclosures

The authors declare that they have no known competing financial interests or personal relationships that could have appeared to influence the work reported in this paper.
